# Supplementary material for: Reconciling Mining with the Conservation of Cave Biodiversity: A Quantitative Baseline to Help Establish Conservation Priorities
Source: PLoS One. 2016 Dec 20;11(12):e0168348. doi: 10.1371/journal.pone.0168348 (PMC5173368; doi:10.1371/journal.pone.0168348)
Supplement: S1 Dataset — (ZIP) [file pone.0168348.s002.zip › Taxa/Serra Norte/SN_2013/N5S/Lista N5S-19.pdf]

CAVIDADE N5S-0019

Classe	Ordem	Fam/Outros	Gên/Outros	Espécie	S-U	U-S	Zon.
Arachnida	Acari	Anystidae	*Erythracarus*	sp.1	X		E
Arachnida	Acari	Trombidiformes		sp.49	X		P
Arachnida	Acari			jovem		X	
Arachnida	Amblypygi	Phrynidae	*Heterophrynus*	*longicornis*	1	X	
Arachnida	Araneae	Ctenidae		jovem		X	
Arachnida	Araneae	Ochyroceratidae	*Ochyrocera*	sp.1	X	X	P
Arachnida	Araneae	Pholcidae		jovem	X		E
Arachnida	Araneae	Theridiosomatidae	*Plato*	sp.1	X	X	E/P
Arachnida	Opiliones	Escadabiidae		sp.2		X	
Arachnida	Opiliones	Neogoveidae		jovem		X	
Arachnida	Palpigradi			jovem		X	
Arachnida	Pseudoscorpiones	Bochicidae		sp.1	X	X	P
Arachnida	Pseudoscorpiones	Chthoniidae	*Pseudochthonius*	sp.2	X	X	P
Arachnida	Pseudoscorpiones	Chthoniidae		sp.		X	
Chilopoda	Scutigeromorpha	Pselliodidae	*Sphendononema*	*guildingii*	2	X	P
Diplopoda	Polydesmida	Pyrgodesmidae		sp.1	X	X	P
Entognatha	Collembola	Paronellidae		sp.1	X		E
Entognatha	Collembola	Paronellidae		sp.4	X		E
Entognatha	Collembola	Sminthuroidea		sp.2	X		P
Entognatha	Collembola			sp.		X	
Entognatha	Diplura	Campodeidae		sp.1	X		P
Insecta	Blattodea			jovem	1		E
Insecta	Coleoptera	Carabidae	*Coarazuphium*	sp.n.1	X		P
Insecta	Diptera	Cecidomyiidae	Cecidomyiinae	sp.	X		E
Insecta	Diptera	Drosophilidae		jovem		X	
Insecta	Diptera	Phoridae	Phorinae	sp.	X		P
Insecta	Diptera	Psychodidae	Phlebotominae	sp.	X		P
Insecta	Diptera	Tipulidae	Tipulinae	sp.	X		E
Insecta	Heteroptera	Dipsocoroidea		sp.	X		P
Insecta	Heteroptera	Reduviidae	Emesinae	sp.	3	X	P
Insecta	Heteroptera	Reduviidae	Reduviinae	sp.	1		E
Insecta	Homoptera	Cixiidae		sp.1	X	X	P
Insecta	Homoptera	Cixiidae		sp.9	X		E
Insecta	Hymenoptera	Formicidae	*Cyphomyrmex*	sp.		X	
Insecta	Hymenoptera	Formicidae	*Eurhopalothrix*	sp.1	X		E/P
Insecta	Hymenoptera	Formicidae	*Hypoponera*	sp.		X	
Insecta	Hymenoptera	Formicidae	*Octostruma*	sp.		X	
Insecta	Hymenoptera	Formicidae	*Pachycondyla*	sp.1	X		P
Insecta	Hymenoptera	Formicidae	*Paratrechina*	sp.1	X		E
Insecta	Hymenoptera	Formicidae	*Paratrechina*	sp.2	X		E
Insecta	Lepidoptera			jovem		X	
Insecta	Orthoptera	Phalangopsidae	*Paraclodes*	jovem	1		E
Insecta	Orthoptera	Phalangopsidae	*Phalangopsis*	sp.	18	X	
Insecta	Psocoptera	Manicapsocidae	*Nothoentomum*	sp.1	X		E
Gastropoda	Pulmonata	Subulinidae	*Lamellaxis*	sp.		X	
Gastropoda	Pulmonata	Systrophiidae	*Happia*	sp.	X		P
Malacostraca	Isopoda	Scleropactidae	*Circoniscus*	*buckupi*	X		P
Malacostraca	Isopoda	Scleropactidae		sp.		X	
Symphyla		Scutigerellidae		sp.	X		P

Amphibia	Anura	Strabomantidae	*Pristimantis*	cf. *fenestratus*	1		E
Mammalia	Chiroptera	Emballonuridae	*Peropteryx*	sp.		X	
Mammalia	Rodentia	Echimyidae	*Proechimys*	sp.		X	
